# Adjuvant radiation improves survival outcomes in adrenocortical carcinoma: A population-based study

**DOI:** 10.1097/MD.0000000000041313

**Published:** 2025-01-24

**Authors:** Kunming Zhan, Xianguo Ruan, Hongtao Jia

**Affiliations:** a Department of Urology, Xiang Yang No.1 People’s Hospital Affiliated Hospital of Hubei University of Medicine, Xiangyang, China; b Department of Urology, Shiyan People’s Hospital, Jinzhou Medical University Training Base, Shiyan, China.

**Keywords:** adjuvant radiation therapy, adrenocortical carcinoma, nomogram, SEER

## Abstract

The aim of this study was to evaluate the clinical benefits and outcomes of adjuvant radiation therapy on adrenocortical carcinoma (ACC) patients. All patients with ACC that were reported between 2010 and 2015 were identified from the Surveillance, Epidemiology, and End Results database. A forward-stepwise Cox proportional hazards regression was used to identify independent risk factors. A new nomogram was created to predict overall and cancer-free survival probabilities at 1, 3, and 5 years for ACC patients. The concordance index, the area under the receiver operating characteristic curve, calibration curves, and decision curve analysis were used to validate the accuracy and reliability of the model. A total of 426 ACC patients were enrolled in this study, of which 84 (19.7%) cases underwent adjuvant radiation therapy (RT). Six factors (age, T stage, type of surgery, radiation, and bone and liver metastases) were significantly related to overall survival (OS) (*P* < .05). Five factors (T stage, type of surgery, radiation, and bone and liver metastases) were significantly related to cancer-specific survival (CSS) (*P* < .05). A nomogram for OS and CSS was constructed. The area under the receiver operating characteristic curve values for 1-, 3-, and 5-year OS were 0.85, 0.818, and 0.814, respectively, and for CSS, they were 0.839, 0.803, and 0.796, respectively. C-indices were 0.756 and 0.749 for OS and CSS, respectively, indicating that the nomograms for OS and CSS had satisfactory discriminative power. A good consistency between the observed and the predicted survival was found in the calibration curves, and the nomogram has a good net clinical benefit, which is presented in the decision curve analysis curves. Our study demonstrates that adjuvant RT can produce a significant improvement in survival outcomes for ACC patients and suggests that adjuvant RT should routinely be applied in ACC patients.

## 1. Introduction

Adrenocortical carcinoma (ACC) is an extremely rare and aggressive endocrine malignancy with an estimated incidence of 0.5 to 2.0 cases per million people annually. ACC accounts for 0.2% of all cancer deaths in the United States.^[1]^ Complete surgical resection is still the only curative treatment for ACC patients; however, locoregional recurrence is extremely high due to its highly aggressive behavior. One-third of ACC patients receiving R0 resection still experience locoregional recurrence.^[2,3]^ Although most ACC patients undergo surgical treatment, the 5-year overall survival (OS) rate only ranges from 15% to 60% due to hormonal hypersecretions, high locoregional recurrence, and high rates of synchronous metastases at initial diagnosis.^[4–6]^

Thus, adjuvant therapy is needed for patients with ACC to improve their survival outcomes; however, adjuvant therapy options, including radiation therapy (RT), chemotherapy, and mitotane, are limited.^[7,8]^ In previous studies, ACC was characterized by a congenital resistance to radiotherapy and chemotherapy. Although mitotane-associated survival benefits for ACC patients are still controversial, mitotane is the only medicine approved by the Food and Drug Administration.^[7–9]^ However, significant toxicity and frequent drug monitoring limit the use of mitotane.^[10]^

In recent years, with the advances in radiotherapy technology, more patients with ACC have undergone RT. Most studies using modern radiation techniques have demonstrated that adjuvant RT can produce significant reductions in locoregional recurrences after complete surgical resection. However, the benefits of RT in facilitating ACC survival are still controversial.^[11–14]^ Wang et al analyzed 583 elderly ACC patients and found only surgery was not performed, and the advanced European Network for the Study of Adrenal Tumors (ENSAT) stage was associated with worse outcomes. RT did not produce the effects on survival outcomes based on the univariate and multivariate survival analyses. However, Nelson et al^[12]^ analyzed 1184 ACC patients and found that adjuvant RT was associated with a 40% decrease in the risk of death after surgical resection, but only 14% of ACC patients were undergoing RT. Recently, a population-based retrospective analysis also demonstrated that adjuvant RT could facilitate improvements in survival in patients with nonmetastatic ACC, especially for those patients with a high risk of recurrence.^[13]^ However, the analysis failed to determine whether adjuvant RT could also facilitate improvements in survival in metastatic ACC patients.

Therefore, in this study, a large population-based data set was used to examine the benefit of adjuvant radiation in the treatment of all-stage ACC patients.

## 2. Materials and methods

### 2.1. Data source and patient selection

**Figure 1.**
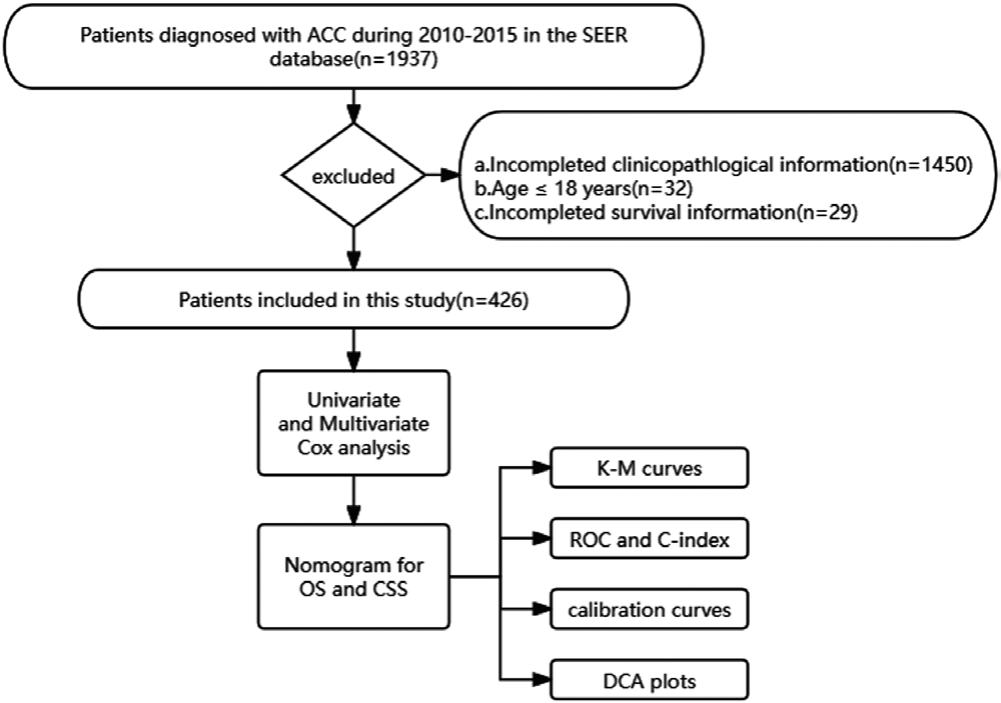
Follow chart of screening process for all patients and study process. ACC = adrenocortical carcinoma, CSS = cancer-specific survival, DCA = decision curve analysis, K-M = Kaplan-Meier, OS = overall survival, ROC = receiver operating characteristic, SEER = Surveillance, Epidemiology, and End Results.

This retrospective cohort was extracted from the Surveillance, Epidemiology, and End Results (SEER) database, which covers data on cancer incidence and survival from 18 population-based registries in the United States using the SEER*STAT (version 8.4.3) software. The third edition of the International Classification of Diseases for Oncology (ICD-O-3) coding system was used for patient selection. Site codes C74.0-C74.9 were used to identify the primary tumor site (adrenal gland) and histological code ICD 8370 for ACC. The flowchart of the patient screening process is shown in Figure 1.

### 2.2. Data collection and outcomes

The clinicopathological factors of ACC patients were extracted as variables for prognostic prediction. Variables included age at first diagnosis, sex, race, marital status, median household income, laterality of the primary tumor, T and N stages, primary site of surgery, chemotherapy, RT, and organ metastases status (bone, liver, and lung). The tumor stage was described based on the seventh edition of the American Joint Committee on Cancer (AJCC).

Overall and cancer-specific survivals (OS and CSS, respectively) were the primary endpoints for survival analysis in this research. OS was defined as the time from the diagnosis of ACC to death caused by any reason, and CSS was defined as the time from the diagnosis of ACC to death due to ACC.

### 2.3. New nomogram construction

The prognostic factors for CSS and OS were identified using a Cox proportional hazard regression. We used the forward-stepwise Cox proportional hazards regression to identify the independent risk factors, after which a new nomogram was constructed based on the independent risk factors to predict OS and CSS at 1-, 3-, and 5-year intervals for ACC patients. The concordance index (C-index) and the area under the receiver operating characteristic curve (AUC) were calculated to evaluate the discrimination and accuracy ability of the new nomogram. Calibration curves were constructed based on 1000 bootstrap samples to evaluate the nomogram-predicted and actual outcomes. Furthermore, a decision curve analysis (DCA) was performed to evaluate the clinical net benefit of the new nomogram.

### 2.4. Statistical analyses

Statistical analyses and figures were evaluated using R-software (version 4.2.2) and IBM SPSS Statistics (version 22). Median values and interquartile ranges were used to reflect continuous variables (age), and frequency was used for categorical variables. We used the Pearson chi-squared and Wilcoxon rank sum tests to compare the 2 groups. The log-rank test and Kaplan-Meier survival curves were used to compare OS and CSS between low- and high-risk groups. A hazard ratio supplemented with a 95% confidence interval was derived based on the univariate and multivariable analyses. All *P* values were obtained from 2-sided tests, and *P* < .05 was considered to represent a significant difference.

## 3. Results

### 3.1. Patient characteristics

A total of 426 ACD patients were enrolled in this study, of which only 84 (19.7%) cases that underwent adjuvant RT were enrolled in this study. Table 1 presents the demographic characteristics of the patients in the 2 groups. The median age of patients in the no-adjuvant RT group was higher than the patients in the adjuvant RT group (58 vs 54.5 years; *P* = .065). The chemotherapy rate was higher in the adjuvant RT group, and a significant difference between the 2 groups was observed (*P* < .001). Patients with bone metastases were more likely to receive adjuvant RT (*P* < .001), whereas patients with lung metastases were less likely to receive adjuvant RT (*P* = .047). No significant difference between the 2 groups in terms of sex, race, median income, marital status, laterality of the primary tumor, T and N stages, primary site of surgery, and liver metastases was found.

**Table 1:** Patients clinical and pathological features between radiotherapy group and nonradiotherapy group.

Characteristics	Nonradiotherapy group (N = 342)	Radiotherapy group (N = 84)	*P* value
Age, median (IQR)	58 (46, 68)	54.5 (44.5, 62)	.065
Sex			.46
Female	211 (61.7%)	48 (57.1%)	
Male	131 (38.3%)	36 (42.9%)	
Race			.62
White	288 (84.2%)	70 (83.3%)	
Black	27 (7.9%)	5 (6.0%)	
Others	27 (7.9%)	9 (10.7%)	
Median income			.90
High	114 (33.3%)	27 (32.1%)	
Low	228 (66.7%)	57 (67.9%)	
Marital			.71
Married	198 (57.9%)	51 (60.7%)	
Unknown/unmarried	144 (42.1%)	33 (39.3%)	
Laterality			.22
Left	186 (54.4%)	39 (46.4%)	
Right	156 (45.6%)	45 (53.6%)	
T stage			.65
pT1–2	186 (54.4%)	42 (50%)	
pT3–4	156 (45.6%)	42 (50.4%)	
N stage			.70
pN0	302 (88.3%)	76 (90.5%)	
pN1	40 (11.7%)	8 (9.5%)	
Surgery performed			.30
No	77 (22.5%)	14 (16.7%)	
Yes	265 (77.5%)	70 (83.3%)	
Chemotherapy			.002
No	195 (57.0%)	32 (38.1%)	
Yes	147 (43.0%)	52 (61.9%)	
Bone metastases			<.001
No	333 (97.4%)	70 (83.3%)	
Yes	9 (2.6%)	14 (16.7%)	
Liver metastases			.87
No	284 (83.0%)	71 (84.5%)	
Yes	58 (17.0%)	13 (15.5%)	
Lung metastases			.047
No	268 (78.4%)	74 (88.1%)	
Yes	74 (21.6%)	10 (11.9%)	

IQR = interquartile range.

### 3.2. Prognostic risk factors for OS and CSS

We used the univariate and multivariate Cox regression models to identify the prognostic risk factors related to OS and CSS. Tables 2 and 3 present the results from the Cox regression model. After the forward-stepwise Cox proportional hazards regression, 6 factors (age, T stage, type of surgery, RT, bone, and liver metastases) were found to be significantly related to OS (*P* < .05), and 5 factors (T stage, type of surgery, radiation, bone, and/or liver metastases) were found to be significantly related to CSS (*P* < .05).

**Table 2:** Univariate and multivariate survival analysis of ACC patients for overall survival.

Characteristic	Univariate analysis			Multivariate analysis		
	HR	95% CI	*P* value	HR	95% CI	*P* value
Age (yr)	1.017	1.009–1.025	<.001	1.016	1.007–1.024	<.001
Sex						
Male	Reference					
Female	0.903	0.805–1.012	.078			
Marital status						
Married	Reference					
Unknown/unmarried	0.984	0.878–1.103	.785			
Median income						
<$7500	Reference					
≥$75,000	1.022	0.907–1.152	.716			
Race						
White	Reference					
Black	0.705	0.504–0.986	.041			
Others	1.250	0.927–1.683	.143			
Laterality						
Left	Reference					
Right	0.945	0.844–1.057	.319			
T stage						
pT1–2	Reference			Reference		
pT3–4	2.203	1.752–2.770	<.001	1.798	1.392–2.321	<.001
N stage						
pN0	Reference					
pN1	2.571	1.855–3.563	<.001			
Surgery performed						
Yes	Reference			Reference		
No	2.246	1.966–2.564	<.001	3.240	2.377–4.416	<.001
Radiation						
Yes	Reference					
No	1.183	1.020–1.372	.026	1.451	1.048–2.009	.025
Chemotherapy						
Yes	Reference					
No	0.886	0.792–.991	.034			
Bone metastases						
Yes	Reference			Reference		
No	0.649	0.519–0.811	<.001	0.486	0.296–0.798	.004
Liver metastases						
Yes	Reference			Reference		
No	0.476	0.413–0.549	<.001	0.550	0.380–.795	.001
Lung metastases						
Yes	Reference					
No	0.598	0.524–0.681	<.001			

ACC = adrenocortical carcinoma, CI = confidence interval, HR = hazard ratio.

**Table 3:** Univariate and multivariate survival analysis of ACC patients for cancer-specific survival.

Characteristic	Univariate analysis			Multivariate analysis		
	HR	95% CI	*P* value	HR	95% CI	*P* value
Age (yr)	1.010	1.002–1.018	.017			
Sex						
Male	Reference					
Female	0.907	0.802–1.026	.907			
Marital status						
Married	Reference					
Unknown/Unmarried	0.981	0.867–1.110	.762			
Median income						
<$7500	Reference					
≥$75,000	1.007	0.884–1.147	.918			
Race						
White	Reference					
Black	0.745	0.520–1.065	.107			
Others	1.185	0.855–1.642	.308			
Laterality						
Left	Reference					
Right	0.930	0.824–1.050	.243			
T stage						
pT1–2	Reference			Reference		
pT3–4	2.330	1.816–2.990	<.001	2.060	1.589–2.671	<.001
N stage						
pN0	Reference					
pN1	2.728	1.933–3.850	<.001			
Surgery performed						
Yes	Reference			Reference		
No	2.341	2.032–2.696	<.001	3.944	2.885–5.390	<.001
Radiation						
Yes	Reference					
No	1.151	0.983–1.348	.081	1.507	1.075–2.114	.017
Chemotherapy						
Yes	Reference					
No	0.822	0.727–0.929	.002			
Bone metastases						
Yes	Reference			Reference		
No	0.615	0.489–0.773	<.001	0.427	0.259–0.704	.001
Liver metastases						
Yes	Reference			Reference		
No	0.458	0.394–0.533	<.001	0.525	0.372–0.740	<.001
Lung metastases						
Yes	Reference					
No	0.551	0.481–0.633	<.001			

ACC = adrenocortical carcinoma, CI = confidence interval, HR = hazard ratio.

### 3.3. Construction of the OS and CSS nomograms

**Figure 2.**
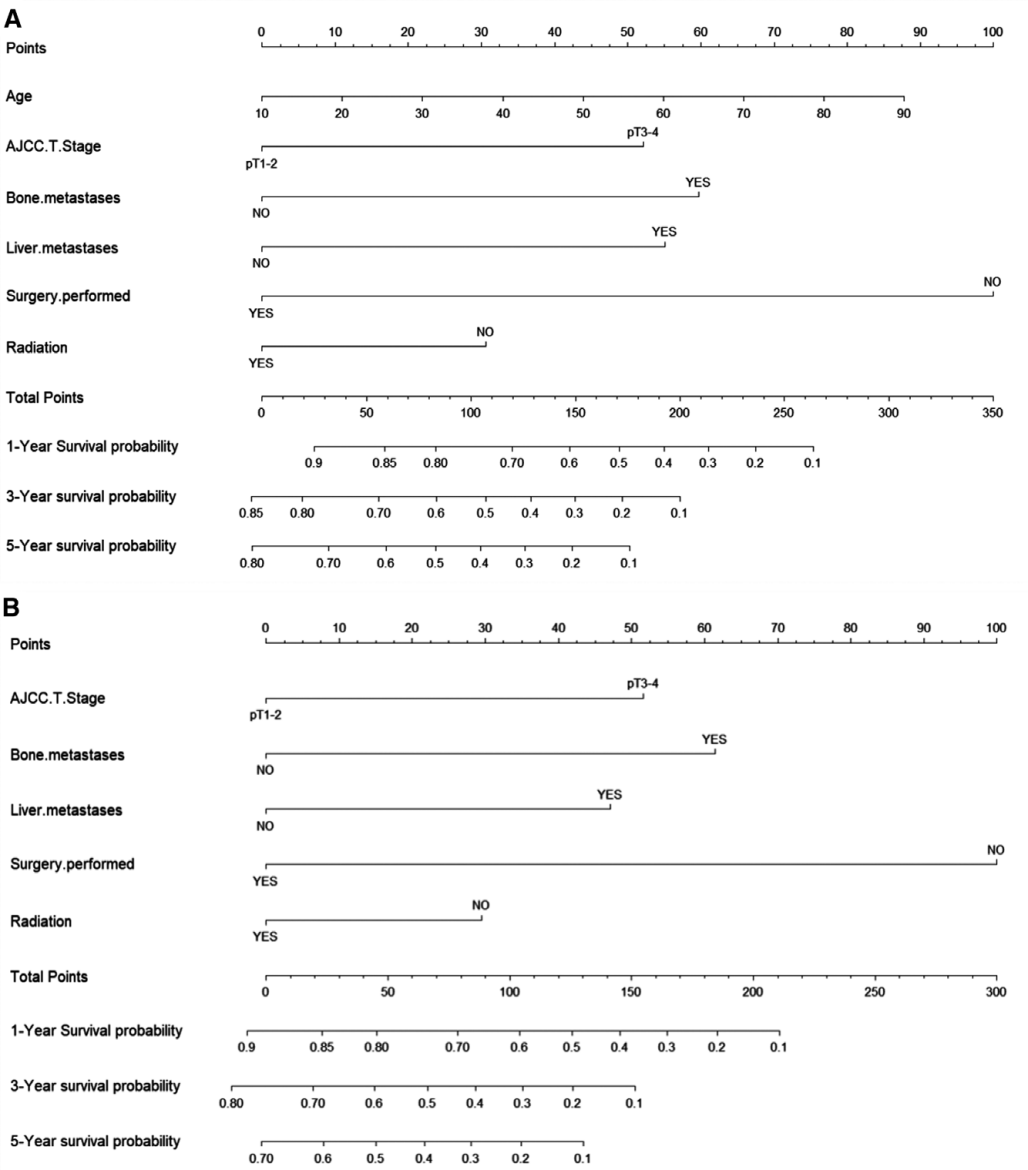
Nomogram for predicting 1-, 3-, and 5-year overall survival (A) and cancer-specific survival (B) for adrenocortical carcinoma patients. AJCC = American Joint Committee on Cancer.

**Figure 3.**
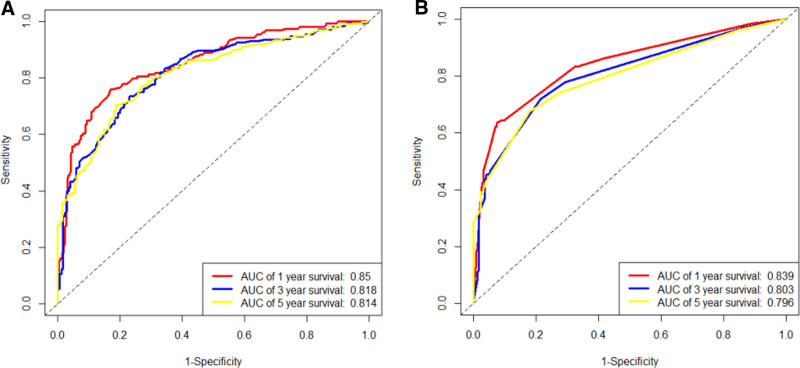
Nomogram receiver operating characteristic curves to predict 1-, 3-, and 5-year overall survival (A) and cancer-specific survival (B). AUC = area under the curve.

**Figure 4.**
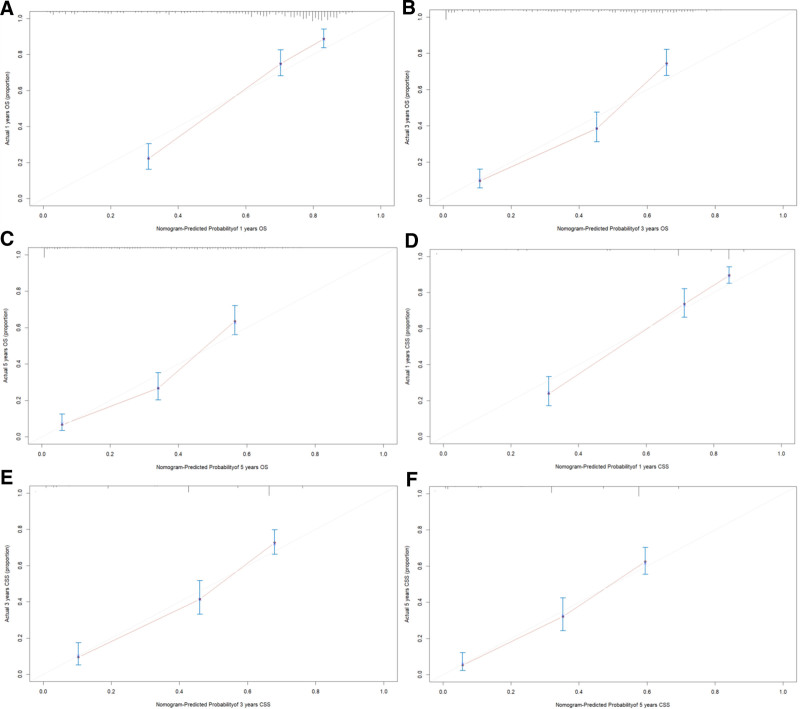
The calibration curves of overall survival (OS) nomogram at 1, 3, and 5 years (A–C). The calibration curves of cancer-specific survival (CSS) nomogram at 1, 3, and 5 years (D–F).

**Figure 5.**
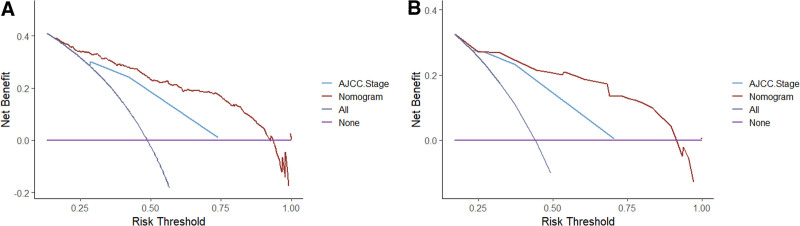
Decision curve analysis predicting overall survival (A) and cancer-specific survival (B). AJCC = American Joint Committee on Cancer.

The prognostic risk factors identified by the Cox regression model were used to construct the nomogram, which can be used to predict the probability of 1-, 3-, and 5-year OS and CSS based on the sum of all scores. The nomograms for predicting OS and CSS of ACC patients were presented in Figure 2. The AUC values were used to validate the accuracy and predictive capabilities of the nomograms. The AUC values for 1-, 3-, and 5-year OS were 0.85, 0.818, and 0.814, respectively; for CSS, the AUC values for 1-, 3-, and 5-year were 0.839, 0.803, and 0.796, respectively (Fig. 3A and B). The C-index was 0.756 for OS and 0.749 for CSS, which showed good discriminatory performance for the model. The survival rate predicted by the nomogram was compared with the actual observation results from the calibration curves (Fig. 4A–F). The nomogram indicated a good net clinical benefit than the AJCC stage model, which is presented in the DCA curves (Fig. 5A and B).

### 3.4. Subgroup survival analyses

**Figure 6.**
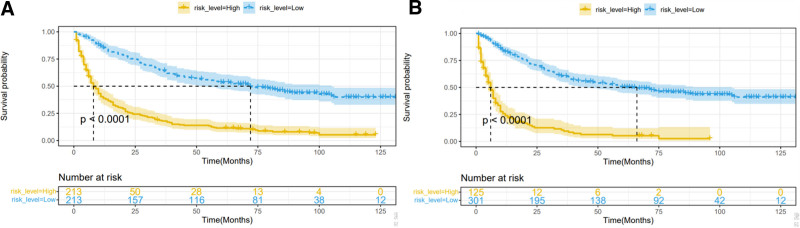
The Kaplan-Meier curves for overall survival (A) and cancer-specific survival (B).

Based on the risk scores calculated by the new nomogram, the median risk scores were used to find the cutoff value after which the patients were divided into low- and high-risk groups. As presented in Figure 6A and B, the patients in the low-risk group had better OS and CSS than those in the high-risk group.

## 4. Discussion

In the present study, population-based data were analyzed to evaluate the benefit of adjuvant RT in the treatment of all-stage ACC patients. We used the univariate and multivariate Cox regression models to identify prognostic risk factors related to OS and CSS and construct a new nomogram to predict the probability of 1-, 3-, and 5-year OS and CSS. We found that adjuvant RT can facilitate a significant improvement in OS and CSS. The new model showed good discriminatory performance and high accuracy. When compared with the AJCC stage model, the nomogram produced a higher net clinical benefit.

ACC is an extremely rare and aggressive malignancy with a low 5-year OS rate. Complete surgical resection is still the only curative treatment, but the locoregional recurrence is extremely high due to its highly aggressive behavior.^[1–6]^ It is challenging to assess the role of adjuvant therapy for this extremely rare disease. Although mitotane is the only medicine approved by the Food and Drug Administration, most mitotane studies were designed as a retrospective series. The clinical benefits of mitotane are controversial, and only the locoregional recurrence-free survival appeared to have improved.^[7–9,15]^ Significant toxicity and frequent drug monitoring also limit the use of mitotane.^[10]^

Adjuvant RT is another treatment option for ACC patients. In studies of some early case series, ACC is characterized by congenital resistance to RT and chemotherapy. Only a few patients receive adjuvant RT, so the clinical benefit of RT in facilitating an improvement in ACC survival is still controversial. In recent years, with the progress of RT technology, more patients with ACC have undergone RT. Most studies using modern radiation techniques have demonstrated that adjuvant RT can lead to significantly reduced locoregional recurrences after complete surgical resection.^[11–14]^ A recent meta-analysis focused on adjuvant RT after surgery for ACC patients also found that adjuvant RT can only produce a reduction in local recurrences. In terms of distant metastases and OS, adjuvant RT was not associated with better outcomes.^[14]^ The largest single institutional study with 424 ACC patients found that adjuvant RT after surgery could facilitate an improvement in local recurrence-free survival. After propensity-matched analysis, it was found that the OS also improved, but only 39 (9.2%) patients had undergone adjuvant RT, which limited the reliability of the conclusions.^[16]^ Another single-institution study included 105 patients with localized ACC and found that postoperative adjuvant RT led to a significant improvement in OS and disease-free survival when compared with surgery alone in ACC patients.^[13]^ In our study, 84 (19.7%) cases underwent adjuvant RT. We also found that adjuvant RT can facilitate improvements in OS and CSS in ACC patients. A new nomogram was constructed to predict the probability of 1-, 3-, and 5-year OS and CSS based on the prognostic risk factors. Patients who did not undergo adjuvant RT had about a 1.5-fold higher risk of CSS death and about a 1.45-fold higher risk of overall death based on a multivariate survival analysis. A recent population-based analysis also constructed a nomogram for elderly ACC patients, but they found that adjuvant RT was not associated with better outcomes. The number of patients who did not undergo adjuvant RT was also limited, and the relatively long timeframe (1975–2016) in this study also produced a reduction in the reliability of the conclusions.^[17]^

Currently, the AJCC and ENSAT staging systems are the most widely used staging systems for ACC patients.^[18–20]^ The accuracies of the 2 staging systems are also controversial. The biggest differences between the 2 staging systems are stages III and IV. In the 2 staging systems, ACC patients with tumors ≤ 5 cm in size without lymph node involvement and distant metastases are defined as stage I. Tumors > 5 cm in size without lymph node involvement and distant metastases are defined as stage II. In the AJCC staging system, stage III includes patients with surrounding tissues or the renal/cava vein invasion without lymph node involvement and distant metastases or with lymph node invasion but without distant metastases. Patients with distant metastases were classified as stage IV. In the ENSAT staging system, stage III only includes patients with surrounding tissues and/or the renal/cava vein invasion without lymph node involvement and distant metastases. Patients with lymph node invasion but without distant metastases were classified as stage IVa according to the number of tumor-involved organs. Stage IV was categorized into stage IVb (3 organs, including N) and stage IVc (>3 organs, including N). Jannello et al tested the accuracy of the ENSAT staging system using the SEER database (2004–2020) and compared the benefits of the 2 staging systems. They found the accuracy of ENSAT is the same as that of AJCC in predicting CSS (74.7% vs 74.5%) and OS (73.7% vs 73.5%).^[21]^ In our study, we compared the clinical benefit between AJCC staging and the new nomogram based on the DCA curves. We found the new nomogram has a better net clinical benefit than the AJCC stage model for OS and CSS in ACC patients.

All stages of ACC patients included in this study were used to evaluate the benefits of adjuvant RT. We found that ACC patients who had undergone adjuvant RT had better survival outcomes. Wu et al analyzed 365 ACC patients in stages I to III, including 55 (15.1%) who had received adjuvant RT after surgery. They found that adjuvant RT was associated with improved survival in patients with nonmetastatic ACC who underwent radical surgery, especially those with a high risk of recurrence.^[22]^ As for stage IV patients, Wu et al also found that adjuvant RT may be associated with improved survival in patients with synchronous metastatic ACC based on a population-based analysis.^[3]^ Given the rare occurrence of the disease, most of the studies in the literature used population-based data to analyze the effectiveness of adjuvant RT. Large-scale randomized controlled trials are needed to accurately test the benefit of adjuvant RT.

Several limitations of our study should be discussed. First, selection bias was not eliminated in our study because of the retrospective nature. Second, some important pieces of information, such as the details of chemotherapy and RT schedules, the extent of surgery, and locoregional recurrence, which may affect the survival of ACC patients, were not included. Finally, multi-centers and large-scale randomized controlled trials are needed, and external validation of our nomogram is also needed to confirm its predictive effect.

## 5. Conclusion

Our study demonstrates that adjuvant RT can significantly facilitate improvements in the survival outcome for ACC patients based on a large national data set, although only a small percentage of patients had undergone adjuvant RT. Therefore, our findings suggest that adjuvant RT should routinely be used in ACC patients. Multi-center and large-scale randomized controlled trials are needed to evaluate the value of adjuvant RT in ACC patients.

## Author contributions

**Data curation:** Kunming Zhan, Xianguo Ruan.

**Methodology:** Kunming Zhan, Xianguo Ruan.

**Software:** Kunming Zhan, Xianguo Ruan.

**Writing – original draft:** Kunming Zhan.

**Investigation:** Xianguo Ruan.

**Project administration:** Xianguo Ruan.

**Resources:** Xianguo Ruan.

**Conceptualization:** Hongtao Jia.

**Formal analysis:** Hongtao Jia.
